# Comparative analysis of fixation techniques for signal detection in avian embryos

**DOI:** 10.1016/j.ydbio.2024.09.002

**Published:** 2024-09-06

**Authors:** Camilo V. Echeverria, Tess A. Leathers, Crystal D. Rogers

**Affiliations:** Department of Anatomy, Physiology, and Cell Biology, University of California, Davis, Davis, CA, USA

**Keywords:** Paraformaldehyde, Trichloroacetic acid, Immunohistochemistry, IHC, HCR, Chicken, Neural crest, Cadherin, Tubulin, SOX, PAX

## Abstract

The choice of fixation method significantly impacts tissue morphology and visualization of gene expression and proteins after *in situ* hybridization chain reaction (HCR) or immunohistochemistry (IHC), respectively. In this study, we compared the effects of paraformaldehyde (PFA) and trichloroacetic acid (TCA) fixation techniques prior to HCR and IHC on chicken embryos. Our findings underscore the importance of optimizing fixation methods for accurate visualization and subsequent interpretation of HCR and IHC results, with implications for probe and antibody validation and tissue-specific protein localization studies. We found that TCA fixation resulted in larger and more circular nuclei and neural tubes compared to PFA fixation. Additionally, TCA fixation altered the subcellular fluorescence signal intensity of various proteins, including transcription factors, cytoskeletal proteins, and cadherins. Notably, TCA fixation revealed protein signals in tissues that may be inaccessible with PFA fixation. In contrast, TCA fixation proved ineffective for mRNA visualization. These results highlight the need for optimization of fixation protocols depending on the target and model system, emphasizing the importance of methodological considerations in biological analyses.

## 1. Introduction

*In situ* hybridization chain reaction (HCR) and immunohistochemistry (IHC) are cornerstone methods to visualize cell and tissue-level phenomena, revealing potential molecular interactions, gene expression, and protein localization within biological specimens. Central to these techniques are the process of fixation, crucial for preserving targets, tissue morphology, and antigenicity. Here, we compare two prevalent fixatives—paraformaldehyde (PFA) and trichloroacetic acid (TCA)—prior to HCR and IHC analyses. The study delves into the respective impacts of each fixative method and time length on tissue-specific signal detection, including cellular morphology and intensity. Our goal is to unravel the nuanced effects on the quality and reliability of HCR and IHC outcomes and potential differences between the two methods. While HCR detects specific expressed genes via probes complementary to the mRNA sequence, IHC results can be variable depending on the tissue sample used, antibody efficacy, and antigen type and localization (Ayoubi et al., 2023). Prior studies identified that specific fixation methods are necessary to visualize proteins that are localized to different sub-cellular regions or cellular structures (Feng et al., 2021; Hua and Ferland, 2017). Through a systematic investigation, we provide comprehensive insights into how the choice of fixative can alter results, which may empower researchers in optimizing signal detection protocols for enhanced accuracy and reproducibility in biological analyses. Specifically, here we analyze the outcomes of fixing wholemount *Gallus gallus* (chicken) embryos with PFA and TCA and use HCR and IHC without antigen retrieval to identify how those methods alter the signal visibility, tissue specificity, and fluorescence intensity of transcripts and proteins that are normally found in the nucleus, cytoplasm, and cell membrane.

In developmental biology, investigating gene expression and protein localization changes in vertebrate embryos using HCR and IHC offers a profound understanding of intricate molecular processes governing embryogenesis. At minimum, both techniques can provide basic details of cell and tissue types in which a gene or protein is expressed, but IHC can also offer insight into dynamic cellular and subcellular localization changes of specific proteins across developmental stages. The selection, specificity, and efficacy of fixation methods and detection tools significantly influences the accuracy and fidelity of developmental studies. Given the delicate nature of embryonic tissues, a multitude of IHC studies in embryonic tissues use aldehyde fixation in the form of formaldehyde, formalin, or PFA (Table 1). PFA is often favored for embryonic specimens due to its ability to cross-link proteins and amines in DNA and RNA, thus preserving tissue architecture and maintaining structural epitopes (Stumptner et al., 2019). Upon contact with tissue, PFA undergoes hydrolysis to form formaldehyde, its active component, and this reactive aldehyde efficiently crosslinks proteins via amino acid bridges (Klockenbusch and Kast, 2010; Nadeau and Carlson, 2007; Solomon and Varshavsky, 1985). The ability of PFA to create stable crosslinks makes it the fixative agent of choice to preserve structural epitopes for subsequent microscopic analysis and downstream experimentation.

Conversely, TCA fixation, known for its permeabilization and dehydration, presents an alternative with potential benefits to access hidden epitopes in embryos but is used less frequently in developmental studies (Lee et al., 2008; Martin et al., 2022). Upon application, TCA penetrates tissues and promptly precipitates proteins by causing their denaturation and aggregation through acid-induced coagulation, which may enhance or deter the ability of antibodies to bind to specific antigens depending on their target (Rajalingam et al., 2009). The acidic nature of TCA and high precipitation capacity result in rapid and robust fixation, preserving tissue architecture by solidifying cellular constituents and preventing enzymatic degradation (Hao et al., 2015; Lakatos and Jobst, 1992). While TCA fixation may alter some protein structures due to its denaturing effects, this effect can be beneficial when used against bulky or hidden epitopes in subsequent histochemical and immunohistochemical analyses.

While mRNA visualization methods have been honed over multiple decades in various species (Biris and Yamaguchi, 2014; Broadbent and Read, 1999; Dworkin-Rastl et al., 1986; Harland, 1991; Hemmati--Brivanlou et al., 1990; Holland, 1999; Hsu and Tuan, 1997; Lecuyer et al., 2008; Luc et al., 2019; Nieto et al., 1996; Wang et al., 2022), visualizing various types of proteins within cells demands a careful approach, considering the diverse subcellular localizations, divergent amino acid sequences, and unique tertiary structures they may possess. For proteins localized to distinct subcellular regions such as the nucleus, cytoplasm, or plasma membrane, fixation methods must cater to the preservation of these specific environments. Fixatives like PFA are adept at maintaining the intricate membranous structures and spatial organization within the cytoplasm or plasma membrane. In addition, for proteins residing in the nucleus, fixation methods that effectively permeate nuclear membranes and preserve nuclear morphology become imperative. Moreover, proteins with intricate tertiary structures, such as those forming multimeric complexes or undergoing post-translational modifications, often necessitate fixation techniques that maintain these delicate interactions. Thus, tailoring fixation methods according to subcellular localization and protein tertiary structure becomes pivotal in accurately visualizing diverse protein populations within cells.

The selection of fixation methods for IHC poses a delicate balance between tissue preservation and antibody penetration. While certain fixatives excel in preserving tissue architecture and antigenicity, their robustness might hinder the penetration of certain antibodies into the tissue, limiting the accessibility to targeted antigens. Conversely, fixation methods optimized for better antibody penetration might compromise tissue integrity and antigen preservation, which can alter the ability to use these tissues for downstream processing. Achieving an optimal equilibrium between these two facets is crucial to ensure comprehensive visualization of antigens within tissues, balancing the preservation of structural integrity with the facilitation of antibody access for accurate and reliable analyses.

With this study, we show the outcomes of PFA versus TCA fixation methods specifically in the context of visualizing avian embryonic development using HCR and IHC, shedding light on their distinct impacts on tissue preservation and signal detection to aid researchers in selecting the most suitable approach for developmental investigations. Here, we identify that TCA fixation methods may be optimal to visualize the signal from cytosolic microtubule subunits and membrane-bound cadherin proteins after IHC, but that TCA is subpar to visualize mRNA signals with fluorescence microscopy after HCR or nuclear-localized transcription factors with IHC. In contrast, PFA fixation provides adequate signal strength for proteins localized to all three cellular regions but is optimal for maximal signal strength of nuclear-localized proteins and for visualization of mRNA signals after HCR.

## 2. Materials and methods

### 2.1. Collection and staging of chicken embryos

Fertilized chicken eggs were obtained from UC Davis Hopkins Avian Facility and incubated at 37°C to the desired stages according to the Hamburger and Hamilton (HH) staging guide. After incubation, embryos were dissected out of eggs onto Wattman filter paper and placed into room temperature Ringer’s Solution. Embryos were then fixed using one of the methods listed below prior to IHC.

### 2.2. Fixation methods

**Fig. 1.**
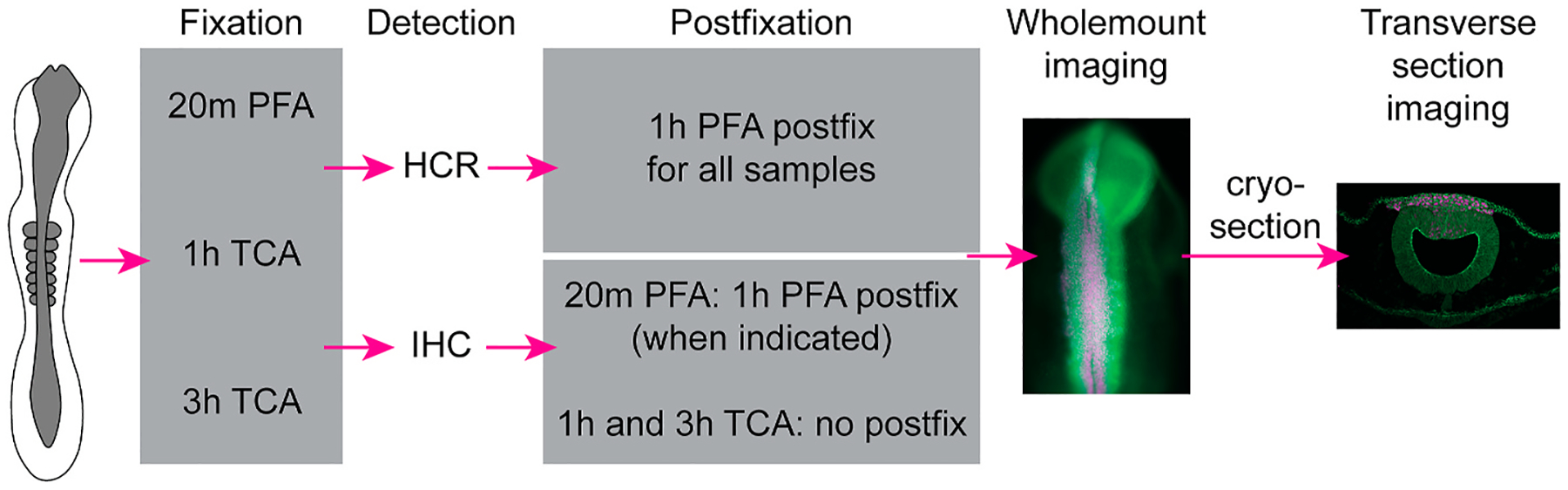
Workflow of fixation and detection methods. Chicken embryos were dissected at desired stages, washed in Ringer’s solution, then fixed in either 2% TCA or 4% PFA. HCR and IHC were then performed as described in the methods. Embryos were either post-fixed for 1h with 4% PFA or not post-fixed, and then imaged in whole mount. All embryos were prepared for cryosectioning using the same methods and then sectioned and imaged in transverse section.

Tissue fixation is described below and the workflow that was used is detailed in Fig. 1.

#### 2.2.1. Paraformaldehyde

Paraformaldehyde (PFA) was dissolved in 0.2M phosphate buffer to make 4% weight per volume (w/v) stock solution, was stored at −20 °C prior to use, and was thawed fresh before use. Embryos were fixed at room temperature with 4% Paraformaldehyde (PFA) for 20 min (20 m). After fixation, embryos were washed in 1X Tris-Buffered Saline (TBS; 1M Tris-HCl, pH 7.4, 5M NaCl, and CaCl_2_) containing 0.5% Triton X-100 (TBST + Ca^2+^) or 1X Phosphate Buffered Saline (PBS) containing 0.1–0.5% Triton X-100 (PBST). Following IHC, 20m PFA-fixed embryos were incubated with and without a 1h postfix in 4% PFA at room temperature to test for differences in tissue structure. Following HCR, all samples were post-fixed for 1h with 4% PFA at room temperature to maintain signal.

#### 2.2.2. Trichloroacetic acid

Trichloroacetic acid (TCA) was dissolved in 1X PBS to make 20% (w/v) stock solution and stored at −20 °C prior to use. It was then thawed and diluted to 2% concentration with 1X PBS fresh before use. Embryos were fixed at room temperature with 2% TCA in 1X PBS for 1h or 3h. After fixation, embryos were washed in TBST + Ca^2+^ or PBST. Following IHC, 1h TCA and 3h TCA-fixed samples were not post-fixed. Following HCR, all samples were post-fixed for 1h with 4% PFA at room temperature to maintain signal.

### 2.3. Fluorescent in situ hybridization chain reaction (HCR)

Fluorescent *in situ* hybridization chain reaction (HCR) was performed using the protocol suggested by Molecular Technologies with minor modifications as described in (Monroy et al., 2022). All probes and kits were acquired from Molecular Technologies. Described briefly, chicken embryos were fixed in 4% PFA for 1h at room temperature or 2% TCA for 1 or 3h at room temperature. Embryos were then washed in PBST and dehydrated in a series of 25%, 50%, 75%, and 100% methanol. Embryos were stored at −20 °C prior to beginning HCR protocol. Embryos were rehydrated in a series of 25%, 50%, 75%, and 100% PBST but were not incubated with proteinase-K as suggested by the protocol. Embryos were incubated with 2.5–10 μL of probes dissolved in hybridization buffer overnight (12–24h) at 37 °C. After washes on the second day, embryos were incubated with 10 μL each of hairpins diluted in amplification buffer at room temperature overnight (12–24h). Embryos were subsequently incubated with 1:500 DAPI in PBST for 1h at room temperature and washed with PBST. All embryos were post-fixed in 4% PFA for 1h at room temperature or 4 °C overnight (12–24h) prior to cryosectioning. Following postfix, embryos were washed in 1X PBS with 0.1% Tween-20 (P-Tween) and imaged in both whole mount and transverse section using a Zeiss Imager M2 with Apotome capability and Zen optical processing software.

### 2.4. Immunohistochemistry (IHC)

**Fig. 2.**
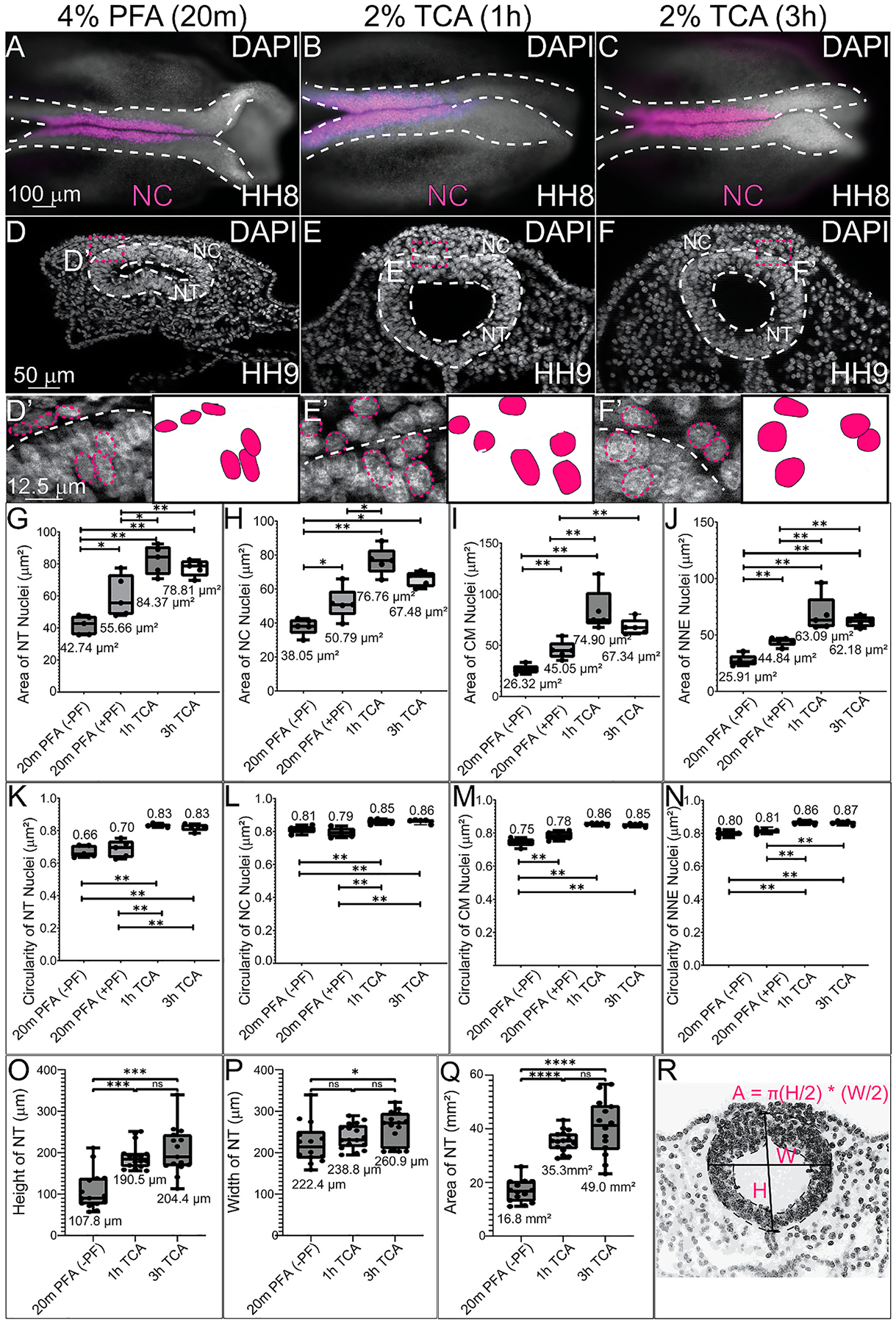
TCA fixation alters NT and nuclear area and circularity. (A–C) Wholemount stage HH8 chicken embryos after IHC for NC marker SOX9 (magenta) and DAPI staining (white). Embryos were fixed in (A, D, D′) 4% PFA for 20m without postfix (B, E, E′) 2% TCA for 1h, or (C, F, F′) 2% TCA for 3h. (D–F) Transverse cryosections from HH9 chicken embryos showing DAPI (white). (D′-F′) High magnification regions from (D–F) with NT outlined in white dashed lines and select nuclei in pink. (G–J) Graphs showing that the area of the NT, NC, CM, and NNC nuclei are significantly different between PFA (with or without post-fixation) and the two TCA fixations. The mean areas of nuclei are shown on graphs. (K–N) Nuclear circularity was measured in the NT, NC, CM, and NNE, and significant differences in circularity were identified between the fixation methods (1.0 is perfect circle). Average circularity measurements are shown on graphs. (O–R) NT height, width, and area were measured and sections from TCA-fixed embryos had significantly larger NT areas than those fixed with PFA (without post-fixation). Mean height, width, and area are shown on graphs. (G–N) For all nuclear area and circularity graphs, n = 5 embryos with 8–12 nuclei measured and averaged for each. (O–Q) For NT height, width, and area graphs, n = 14, 17, and 15 for PFA, 1h TCA, and 3h TCA, respectively. One-way ANOVA with the Mann-Whitney test was used to determine the statistically significant differences between each treatment. Significance values of *, **, ***, and **** indicate P ≤ 0.05, 0.01, 0.001, 0.0001, respectively. Not significant is ns. The scale bar for the wholemount images is 100 μm, the transverse sections is 50 μm, and the high magnification transverse section is 12.5 μm.

**Fig. 3.**
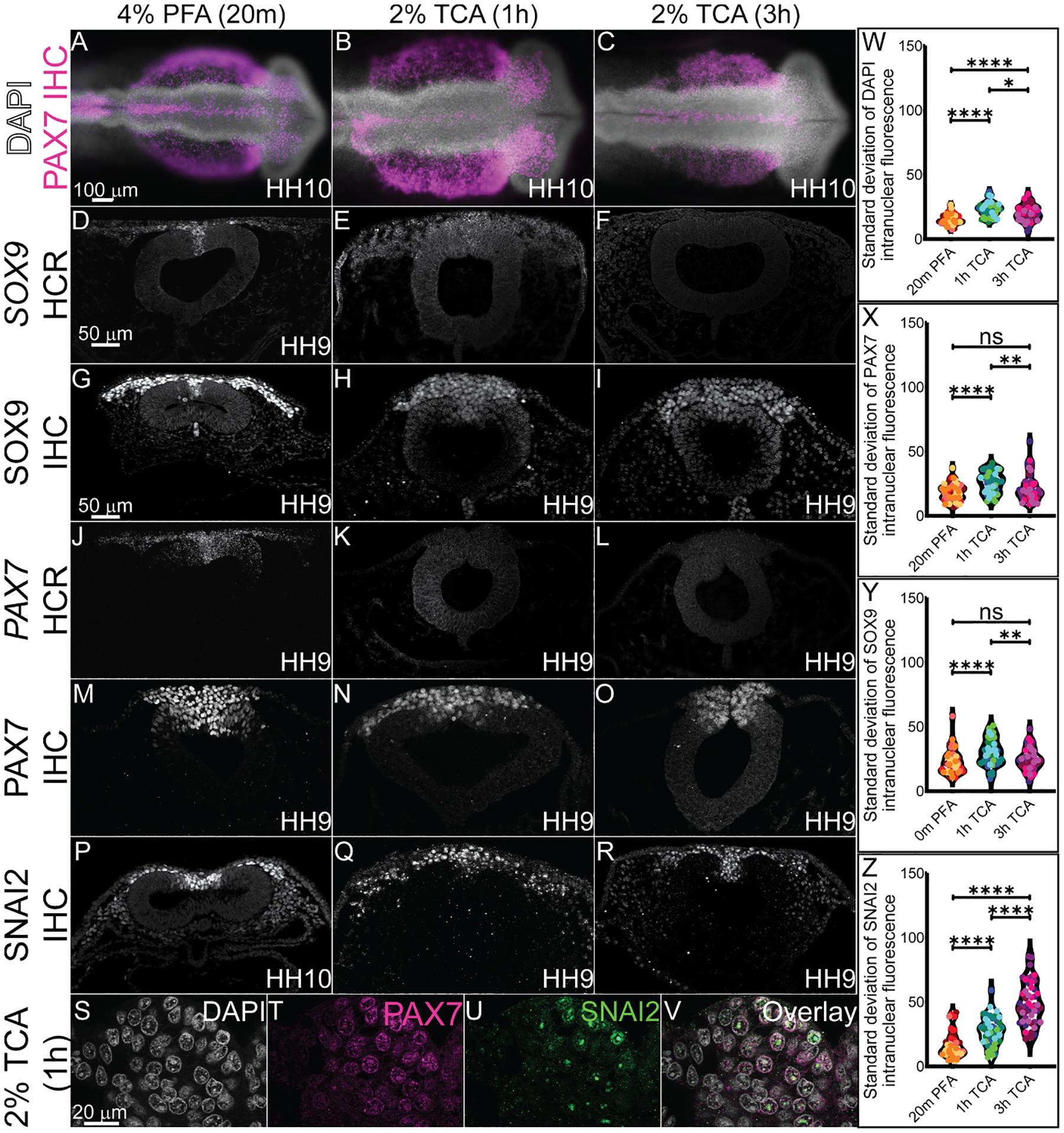
TCA and PFA fixation alter NC-specific transcription factor fluorescence levels across nuclei. HCR and IHC of definitive NC cell markers SOX9, PAX7, and SNAI2 in stage HH9–10 chicken embryos fixed in (A, D, G, J, M, and P) 4% PFA for 20m, (B, E, H, K, N, and Q) 2% TCA for 1h, and (C, F, I, L, O, and R) 2% TCA for 3h. (A–C) Wholemount chicken embryos after all three fixation methods and IHC for PAX7 (magenta) and DAPI staining (white). (D–V) Transverse cryosections from HH9–10 embryos. Transverse sections from embryos after HCR for (D–F) *SOX9* and (J–L) *PAX7*, and IHC for (G–I) SOX9, (M–O) PAX7, and (P–R) SNAI2. (S–V) High magnification images of 2% TCA 1h fixed embryo nuclei after IHC for PAX7 (magenta) and SNAI2 (green) with DAPI staining (white). (W–Z) Violin plots showing standard deviation of (W) DAPI, (X) SOX9, (Y) PAX7, and (Z) SNAI2 fluorescence across the nucleus comparing the three fixative conditions with measurements taken from n = 5 embryos, 10 nuclei from each. Data points from the same individual are plotted with the same color. (W–Z) All plots show a significant difference in the standard deviation of intranuclear fluorescence between 20m PFA and 1h TCA, though only DAPI and SNAI2 show a significant difference between 20m PFA and 3h TCA. The lower standard deviation indicates more diffuse fluorescence across the nucleus while higher standard deviation indicates more punctate fluorescence. Significance values of *, **, ***, and **** indicate p ≤ 0.05, 0.01, 0.001, 0.0001, respectively. Not significant is ns. The scale bar for the wholemount is 100 μm, the transverse section is 50 μm, and the high magnification transverse section is 20 μm.

**Fig. 4.**
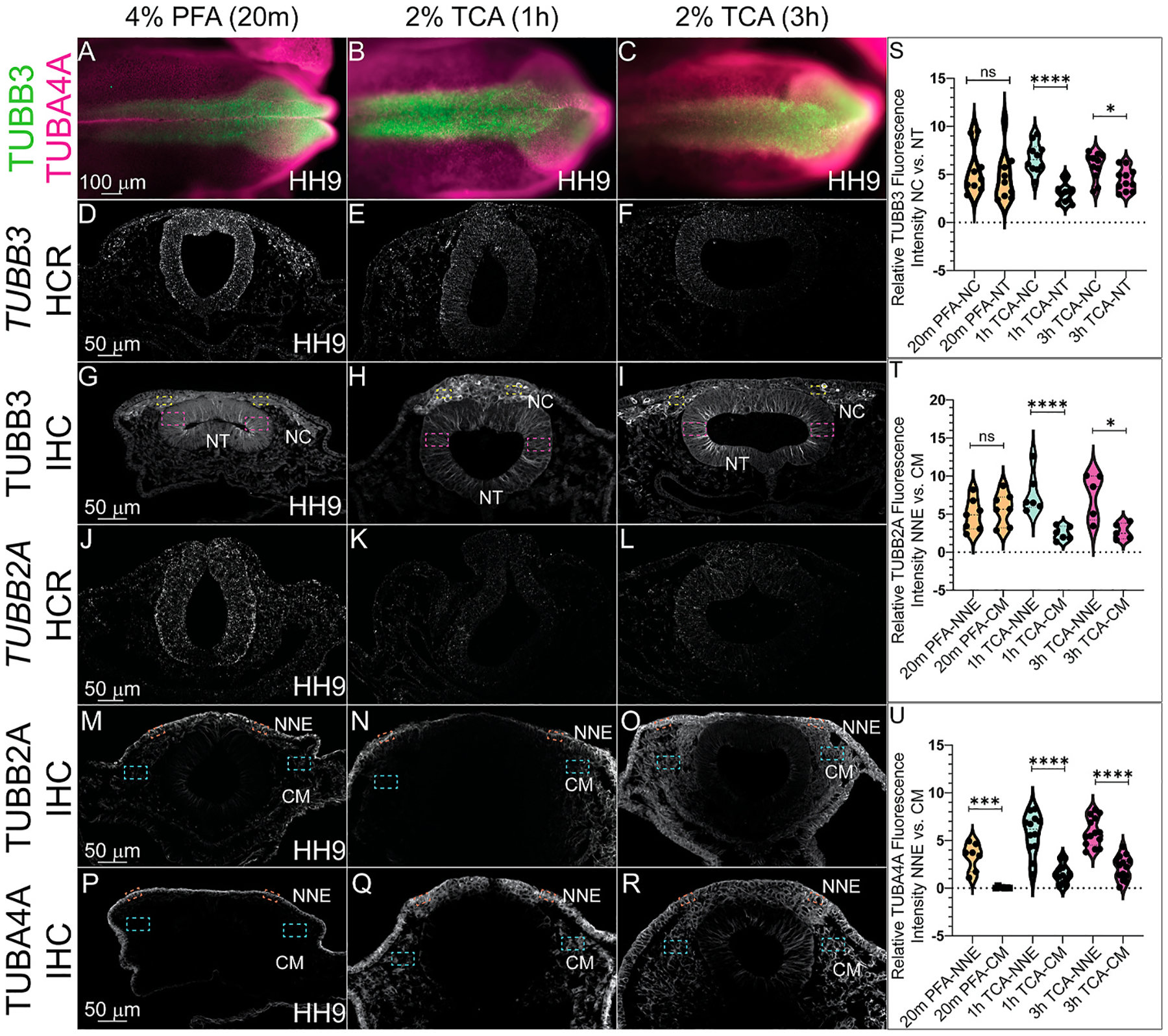
Differences in tissue-specific fluorescence levels of cytoskeletal proteins after TCA fixation. IHC using antibodies against the tubulin isotypes (A-C, G-I) TUBB3, (M–O) TUBB2A, and (A-C, P-R) TUBA4A at HH9 in (A–C) wholemount embryos and (G-I, M-R) transverse cryosections from HH9 embryos. HCR was performed using probes for (D–F) *TUBB3* and (J–L) *TUBB2A*. The embryos were fixed in (A, D, G, J, M, and P) 4% PFA, in (B, E, H, K, N, and Q) 2% TCA for 1h, and in (C, F, I, L, O, and R) 2% TCA for 3h. (A–C) In whole embryos across the three conditions, TUBB3 (green) is visible in the cranial dorsal side of the chicken embryo, and TUBA4A (magenta) across the ectoderm. (D-F, J-L) *TUBB3* and *TUBB2A* mRNA signals are more detectable in PFA-fixed sections. (G–I) IHC for TUBB3 after PFA and TCA fixation. (M–O) IHC for TUBB2A after PFA and TCA fixation. (P–R) IHC for TUBA4A after PFA and TCA fixation. (S) Graph showing fluorescence intensity of TUBB3 in selected tissues (NC and NT) across all three fixative methods (20m PFA in orange, 1h TCA in green, and 3h TCA in magenta). TUBB3 fluorescence was significantly higher in NC cells compared to NT cells after 1h (p ≤ 0.0001, n = 9) and 3h (p ≤ 0.05, n = 10) TCA fixation than it was after PFA fixation. (T) Graph showing fluorescence intensity of TUBB2A in selected tissues (NNE and CM). TCA fixation significantly increased TUBB2A intensity in the CM compared to the NNE in both 1h (p ≤ 0.0001, n = 7) and 3h (p ≤ 0.05, n = 5) TCA treatments compared to PFA fixation. (U) Graph showing fluorescence intensity of TUBA4A in selected tissues (NNE and CM). There were significant differences between NNE and CM intensity in all fixatives, PFA (p ≤ 0.001, n = 7), 1h (p ≤ 0.0001, n = 12) and 3h (p ≤ 0.0001, n = 14) TCA-fixed embryos. NC = neural crest, NT = neural tube, NNE = non-neural ectoderm, CM = cranial mesenchyme. One-way ANOVA with the Mann-Whitney test was used to determine the significance differences in fluorescence intensity. Significance values of *, **, ***, and **** indicate p ≤ 0.05, 0.01, 0.001, 0.0001, respectively. Not significant is ns. Dashed boxes indicate representative locations used to measure fluorescence intensity in different tissues. Yellow is NC, pink is NT, blue is CM, and orange is NNE. The scale bar is marked in the first whole embryo and transverse section of the figure.

**Fig. 5.**
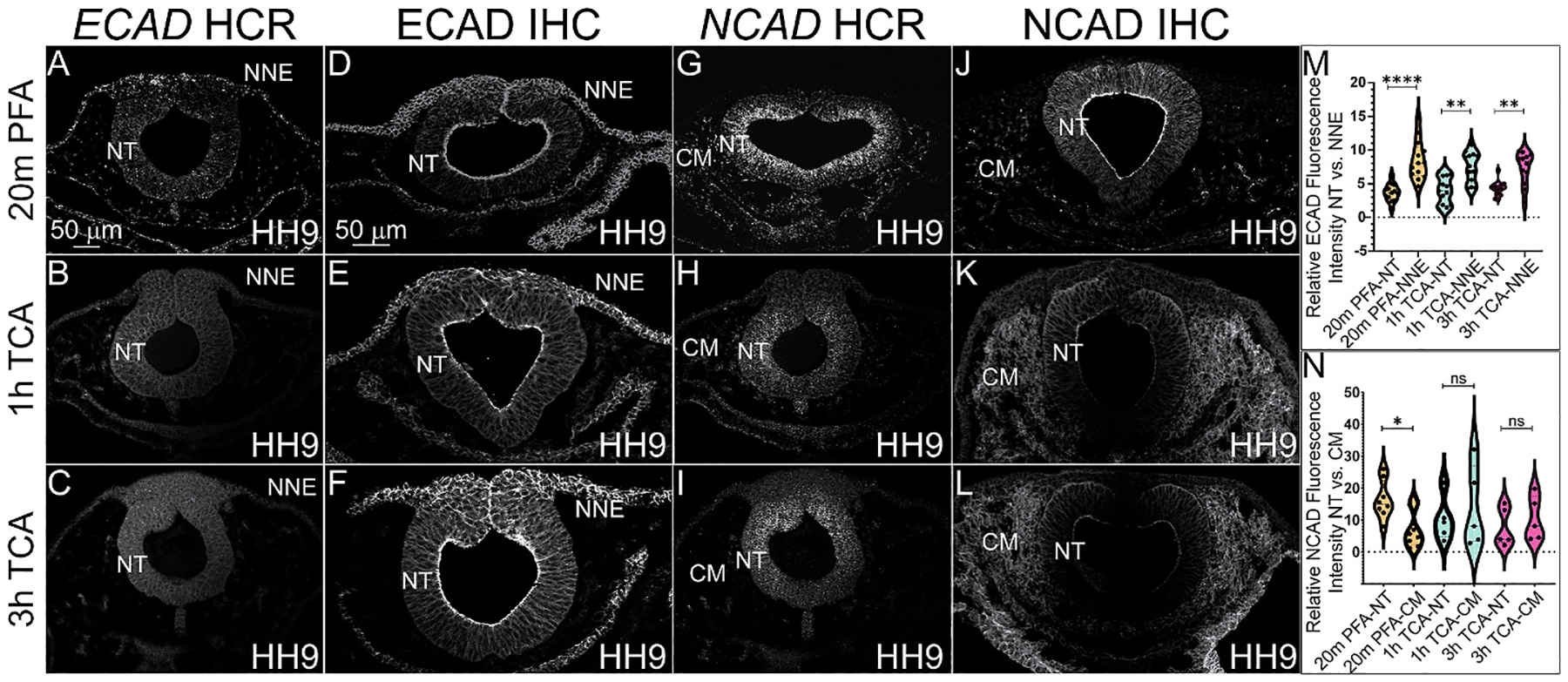
TCA and PFA fixation result in tissue-specific differences in fluorescence intensity of cadherin proteins. (A–L) Transverse cryosections from HH9 chicken embryos. (A–C) Transverse sections comparing three fixative conditions prior to HCR *ECAD* and (D–F) IHC of ECAD. (G–I) HCR for *NCAD* and (J–L) IHC for NCAD in chicken embryos at stage HH9. (A–C) *ECAD* mRNA signal was only detected in embryos fixed with PFA. (D–F) IHC for ECAD of embryos fixed in (D) 4% PFA, (E) 1h TCA, and (F) 3h TCA shows a higher difference between NT and NNE signal after PFA fixation. (G–I) HCR for *NCAD* detected signal after all three fixative methods, but signal intensity was stronger using PFA. (J–L) IHC for NCAD of embryos fixed in (J) 4% PFA, (K) 1h TCA, and (L) 3h TCA shows increased CM and reduced NT signal intensity after TCA fixation. (M) Graph showing ECAD fluorescence intensity in the NNE and NT in embryos fixed in 20m PFA (n = 9, orange), 1h TCA (n = 9, blue) or 3h TCA (n = 5, magenta) fixation. (N) Graph showing NCAD fluorescence intensity in the NT and CM in embryos fixed in 20m PFA (n = 6), 1h TCA (n = 5) or 3h TCA (n = 5). NT = neural tube, NNE = non-neural ectoderm, CM = cranial mesenchyme. One-way ANOVA with the Mann-Whitney test was used for analysis. Significance values of *, **, ***, and **** indicate p ≤ 0.05, 0.01, 0.001, 0.0001, respectively. Not significant is ns. The scale bar for all HCR transverse sections is 50 μm as marked in A and the scale bar for all IHC transverse sections is 50 μm as marked in D.

After fixation, embryos were washed with PBST or TBST + Ca^2+^ and wholemount IHC was performed. To block against non-specific antibody binding, embryos were incubated in PBST or TBST + Ca^2+^ containing 10% donkey serum (blocking solution) for 1h at room temperature or overnight (12–24h) at 4 °C. Primary antibodies were diluted in blocking solution at indicated dilutions (Table 2) and embryos were incubated in primary antibodies for 72–96h at 4 °C. Multiple antibodies from the study have previously been validated in cell lines or chicken embryos. After incubation with primary antibodies, whole embryos were washed in PBST or TBST + Ca^2+^, then incubated with AlexaFluor secondary antibodies diluted in blocking solution (1:500) overnight (12–24 h) at 4 °C. TCA-fixed embryos were then washed in PBST or TBST + Ca^2+^ as the final step before imaging. PFA-fixed embryos had the same final wash with PBST or TBST + Ca^2+^ after secondary incubation and were either immediately imaged (Figs. 2–4) or post-fixed with 4% PFA for 1h at room temperature and washed again with PBST or TBST + Ca^2+^ before imaging (Fig. 5).

### 2.5. Cryosectioning

Following whole embryo imaging, embryos were prepared for cryosectioning by incubation with 5% sucrose in PBS (30m to 1h at room temperature or overnight at 4 °C), followed by 15% sucrose in PBS (3h at room temperature to overnight at 4 °C), and then in 10% gelatin with sucrose in PBS for 3h to overnight at 38–42 °C. Embryos were then flash frozen in liquid nitrogen and were sectioned in an HM 525 NX Cryostats, Epredia, Richard-Allan Scientific in 16 μm sections.

### 2.6. Microscopy

Fluorescence images were taken using Zeiss ImagerM2 with Apotome.2 and Zen software (Karl Zeiss). Whole embryos were imaged at 10X (Plan-NEOFLUAR 10X/0,3 420340–9901) and transverse sections were imaged at 20X (Plan-APOCHROMAT 20X/0,8 420650–9901) with Apotome optical sectioning. Exposure times varied for samples. All images were captured at maximum light intensity and exposure time was adjusted for the strength of each sample signal. DAPI with IHC image exposure times ranged for strongest signal to weakest from (80 ms–3.2 s) and for DAPI with HCR samples, which had significantly lower signal, exposure times ranged from (50 ms–8.3 s). DAPI signal is always the strongest signal with shortest exposure time. Images were adjusted for brightness and contrast uniformly across the entire image in Adobe Photoshop in accordance with journal standards.

### 2.7. Intranuclear fluorescence standard deviation

To find the pixel intensity from one side of the nuclear membrane to the other, 10 nuclei from 5 different embryos per marker (50 total nuclei per fixative for each marker) were analyzed using NIH ImageJ/Fiji with the Dynamic ROI Profiler plugin. These measurements were performed on images converted to grayscale with manual brightness and contrast adjustments through Photoshop. The standard deviation of each set of intranuclear fluorescence measurements was calculated and these data points were plotted as a violin plot with the color representing the chicken embryo the measurement came from (Fig. 3W–Z). Mann-Whitney tests were performed to compare the standard deviation of intranuclear fluorescence across the treatments.

### 2.8. Nuclei and neural tube measurements

#### 2.8.1. Nucleus area and circularity

To quantify the differences in cell area and circularity, nuclei from cells in the neural tube (NT), neural crest (NC), non-neural ectoderm (NNE), and cranial mesenchyme (CM) regions were outlined using Adobe Photoshop and assessed for both area and circularity. From each transverse section, four nuclei were outlined, two from the right side and two from the left side of the embryo. These measurements were done with 2–3 sections per individual, and at least 5 embryos per treatment were measured for each tissue type. Using the Mann-Whitney *U* test to compare the anatomical differences between the TCA and PFA-fixed samples identified that the nuclei of all cell types analyzed had significantly larger areas and were more circular after TCA fixation. The formula for circularity is 4π (area/perimeter^∧^2). A value of 1.0 indicates a perfect circle.

#### 2.8.2. Neural tube height, width, and area

Transverse cryosections were imaged at 20X with the Zeiss Imager. M2 with Apotome and the scale bar was added using the Zeiss Zen software. The neural tube size, height, and width were obtained using ImageJ/Fiji. Using the scale obtained from each sectioned image, a global scale was set to measure the height and width of the neural tube (220 pixels/50 μm) in individual sections from multiple embryos at the same midbrain axial level (n = 14, 17, and 15 for PFA, 1h TCA, and 3h TCA, respectively). The height was obtained using the ImageJ Straight tool by measuring the basal-to-basal distance from the dorsal region of the neural tube to the ventral side. The width was obtained by measuring the basal-to-basal distance of the left and right lateral sides of the neural tube. The overall area of the neural tube was calculated using the formula for the area of an oval (A = π* (height/2)*(width/2) and these measurements were used to compare the neural tube between the three fixative conditions.

### 2.9. Fluorescence intensity analysis

Fluorescence intensity in Figs. 4 and 5 was quantified using NIH ImageJ/Fiji by averaging the relative intensity of tissue-specific regions in section images of chicken embryos. Sections were converted to grayscale, and contrast was adjusted uniformly for each section using Adobe Photoshop. The grayscale images were analyzed using the rectangle tool to quantify the differences in fluorescence between the most visually different tissues for a given marker. The cell type regions analyzed included neural crest (NC) cells, neural tube (NT) cells, non-neural ectoderm (NNE) cells, and cranial mesenchyme (CM) cells. For intensity values, at least 4 regions were sampled from 2 to 6 different cryosection images from each embryo and the average relative fluorescence intensity from each embryo are reported on the graphs as points. Each graph shows relative fluorescence measurements from 5 to 14 individual embryos. The rectangle tool was used to draw a box that was dragged within the image to measure the fluorescence of a tissue region of interest on the right side, then left side of the image, resulting in two images for a given tissue type. This box was then used to measure the fluorescence of the compared tissue type. Between each of these four measurements, the background fluorescence was measured for normalization. The area of the box was between 0.133 and 1.0 pixels^2^, but always the same size within the same image. The measurements obtained through ImageJ/Fiji included the “area,” “area of integrated intensity,” and “mean grey value.” The corrected total cell fluorescence (CTCF) was calculated by subtracting the “area integrated density” from the product of the “area” of a selected region of interest and the “mean gray value” of the background, averaged out the values obtained from each region, then graphed. Each dot on the graphs represents the average of measurements from 1 to 3 cryosection images from 5 to 10 embryos. Number of embryos is indicated in each figure legend.

## 3. Results

### 3.1. TCA fixation alters tissue and nuclear morphology compared to PFA

To identify if different fixation methods affected the general tissue structure, we tested the various methods (4% PFA for 20m with and without a 1h post-fixation after IHC, 2% TCA for 1h and 3h) in Hamburger Hamilton stage 8–10 (HH8–10) chicken embryos. Embryos were collected as described in the methods and fixed in their respective fixatives for 20m, 1h, or 3h (Fig. 1). Embryos were then imaged in whole mount (Fig. 2A–C) and transverse section (Fig. 2D–F, D’–F’). After fixation, HCR or IHC was performed using the antibodies in Table 2 and embryos were stained using the nuclear DNA stain, 6-diamidino-2-phenylindole (DAPI).

To quantify the differences in nuclear area and circularity, nuclei from cells in the NT, NC, NNE, and CM regions were assessed. Use of the Mann-Whitney *U* test to compare the anatomical differences between the TCA and PFA-fixed samples identified that the nuclei of all cell types analyzed had significantly larger areas and were more circular after TCA fixation compared to PFA with or without post-IHC fixation (Fig. 2G–N). Compared to 4% PFA fixation without post-fix, 1h 2% TCA fixation in HH8-HH9 embryos resulted in nuclei with a larger average area in the NT (197% larger, p ≤ 0.01), NC (201% larger, p ≤ 0.01), CM (284% larger, p ≤ 0.01), and NNE (243% larger, p ≤ 0.01) (Fig. 2G–J). Nuclei circularity was measured and nuclei from embryos fixed in 2% TCA were significantly rounder than those fixed with 4% PFA. Compared to 4% PFA fixation without post-fix, 1h 2% TCA fixation in HH8-HH9 embryos resulted, on average, in more circular nuclei in the NT (125%, p ≤ 0.01), NC (104%, p ≤ 0.01), CM (115%, p ≤ 0.01), and NNE (108%, p ≤ 0.01) (Fig. 2K–N). PFA with post-fix averages were more like TCA-fixed nuclei than PFA without post-fix for some tissues but the post-fixation did not fully rescue the significant differences in nuclei area or circularity (Fig. 2G–N). In NT cells, the PFA-fixed nuclei with and without post-fixation had an average circularity score of 0.68, while the TCA-fixed cells had scores of 0.83, with 1.0 indicating a perfect circle (Fig. 2K). In NC cells, the PFA-fixed nuclei had an average circularity score of 0.80, while the TCA-fixed cells had an average score of 0.855 (Fig. 2L). These morphological changes supported our observation that nuclear staining in the NT and NC regions appeared more diffuse in 2% TCA-fixed samples compared to 4% PFA fixation (Figs. 2 and 3).

To determine if the expanded cell nuclei were indicative of generalized changes in tissue structure or morphology, we measured the height, width, and total area of the NT from dorsal to ventral and basolateral to basolateral (Fig. 2O–R). We identified that indeed, on average, fixation with 2% TCA expanded the height, width, and total area of the NTs. Specifically, 1h 2% TCA fixed NTs were 176% taller (p ≤ 0.001) and had a 210% larger area (p ≤ 0.0001) while 3h 2% TCA fixed NTs were 189% taller (p ≤ 0.001), 117% wider (p ≤ 0.05), and had a 291% larger area (p ≤ 0.0001) than PFA fixed NTs.

### 3.2. PFA fixation alters nuclear protein signal detection

PFA is the primary mode of fixation in avian embryos prior to performing HCR and IHC and it works effectively with short fixation times (Table 1). To determine the effectiveness of TCA fixation for mRNA detection using HCR or for use of antibodies targeted to antigens in the nucleus, we used previously characterized antibodies against transcription factors paired box protein 7 (PAX7), SRY-Box 9 (SOX9), and Snail Family Repressor 2 (SNAI2) (Monroy et al., 2022). At HH10, the TCA-fixed wholemount embryos appeared larger than those fixed in PFA (Fig. 3A–C), which is supported by our analyses of NT area (Fig. 2O–R). TCA fixation prior to HCR to visualize gene expression did not work effectively to preserve the mRNA. Compared to the robust and specific expression of *SOX9* (Fig. 3D) and *PAX7* (Fig. 3J) that is visible after PFA fixation, the signals were virtually undetectable using our imaging methods after TCA fixation despite post-fixation after probe amplification (Fig. 3E, F, K, L).

At the protein level, SOX9, PAX7, and SNAI2 fluorescence was robust and appeared pan-nuclear after PFA fixation (Fig. 3G, M, and P). However, although the appearance of these markers in wholemount did not appear markedly different in embryos that were PFA or TCA-fixed, in section, SOX9 and PAX7 expression appeared diffuse, the signal was weaker, and exposure times were longer to capture the signal after TCA fixation (Fig. 3H, I, N, O). In contrast, the SNAI2 signal became more punctate and had variable intensity within each nucleus in TCA fixation compared to a more uniform fluorescence in PFA fixation (Fig. 3, compare P to Q and R). In higher magnification images of sections from TCA-fixed embryos, the DAPI stain overlaps with diffuse PAX7 protein signal, but SNAI2 protein signal appears limited within the nucleus in all NC cells in which it is expressed (Fig. 3S–V). We quantified fluorescent signal across the nuclei, and identified that in fact, there are significant differences in standard deviation of the intranuclear fluorescence in PFA versus TCA-fixed samples, indicating diffuse versus punctate signal fluorescence depending on the type of fixative and the time of fixation (Fig. 3W–Z). In chicken embryos, PFA is our preferred fixation method prior to IHC for robust fluorescence using antibodies against the transcription factors that were evaluated.

### 3.3. Different fixation methods alter the signal intensity of microtubule subunit proteins

To determine how fixation methods affect cytoplasmic and cytoskeletal protein signal, we assessed the various fixation treatments in HH9 chicken embryos and performed HCR and IHC for tubulins (Fig. 4A–R). To identify the effectiveness of PFA and TCA fixation for signal detection of these factors, we used probes against Beta III Tubulin (*TUBB3*) and Tubulin Beta 2A (*TUBB2A*), and antibodies against TUBB3, TUBB2A and Tubulin Alpha 4a (TUBBA4A). Similar to our assessment of mRNA encoding nuclear proteins, we were unable to detect robust gene expression for either *TUBB3* or *TUBB2A* after TCA fixation although the signal was detectable after PFA fixation (Fig. 4D–F, J–L). We concluded similarly that TCA fixation is not effective prior to HCR.

In contrast, signal for all three proteins was visible in all three fixative treatments. TCA fixation appeared to alter the tissue-specific proportional signal brightness compared to PFA fixation for tubulin proteins with IHC. We identified that TUBB3 protein showed stronger fluorescence intensity in NC cells compared to the NT signal after 2% TCA (1h or 3h) versus 4% PFA fixation (Fig. 4G–I, S). The strongest NC-specific TUBB3 signal appeared at 1h 2% TCA fixation (Fig. 4H, S, p ≤ 0.0001). For TUBB2A, with PFA fixation, the protein signal was strongest in the NNE and CM with weaker expression in the NC, and NT (Fig. 4M). With TCA fixation, the TUBB2A fluorescence in the NNE and CM increased compared to the signal in the NC and NT to the point that signal is almost imperceptible in the NT (Fig. 4N and O). However, the NNE signal was significantly stronger than the CM signal after TCA fixation at 1h and 3h (p ≤ 0.0001 and 0.05, respectively). After PFA fixation, TUBA4A signal appears to be solely in the NNE, but after TCA fixation, the protein is visible in the CM as indicated by the increased relative signal intensity, but the NNE signal remains significantly stronger in the NNE than the CM across all fixatives (Fig. 4P–R, U, p ≤ 0.001 for both).

To quantify differences in tissue-specific fluorescence intensity after different fixations, we measured fluorescence intensity in specific tissues and fold changes from the “brightest” signal to the weaker signal. These analyses showed that TUBB3 fluorescence was significantly higher in NC cells compared to NT cells after 1hr and 3h TCA fixation than it was after PFA fixation (Fig. 4S, p ≤ 0.0001, n = 9, and p ≤ 0.05, n = 10). In addition, TCA fixation significantly increased the differences between TUBB2A intensity in the CM compared to the NNE in both 1h and 3h treatments compared to PFA fixation (Fig. 4T, p ≤ 0.0001, n = 7, and p ≤ 0.05, respectively, n = 5). The signal for TUBA4A appeared to be most visible in the NNE after PFA fixation (p ≤ 0.001, n = 7). In the 1h and 3h TCA fixation, the NNE and CM fluorescence signal intensities both increased, but the NNE signal was still significantly stronger than that of the CM (Fig. 4U, p ≤ 0.0001, n = 12, and p ≤ 0.0001, n = 14). These data show that fixation methods can alter the apparent signal intensities in specific tissues.

### 3.4. Different fixation methods affect cadherin protein tissue-specific signal intensity

The localization of N-cadherin (NCAD) and E-cadherin (ECAD) have previously been characterized in chicken embryos across stages using PFA fixation (Dady et al., 2012; Rogers et al., 2018). To determine if TCA fixation is also an efficient method to use prior to HCR or IHC to visualize these genes and proteins, we evaluated the various fixation treatments in HH9 chicken embryos prior to HCR or IHC with antibodies against the two type-I cadherins. Similar to the prior analyses, TCA fixation is not effective to visualize *ECAD* gene expression compared to PFA fixation (Fig. 5A–C). Both TCA fixations prevented the detection of any signal (Fig. 5B and C). *NCAD* is robustly expressed in the NT and can be visualized after both PFA and TCA fixations (Fig. 5G–I). In contrast to all other probes that we tested, we were still able to detect *NCAD* expression in the NT after TCA fixation although the signal was weaker (Fig. 5H and I).

After PFA fixation, both ECAD and NCAD protein signals are visible in the NT at HH9, but while ECAD signal also appears in delaminating NC cells and NNE, the NCAD signal is not detectable in these tissues and instead is visible in the CM confirming previously published results (Fig. 5D–F, J–L) (Dady et al., 2012; Rogers et al., 2018). In 2% TCA at both 1h and 3h fixations, the ECAD signal remains in the same tissues (Fig. 5E and F). We measured the relative fluorescence intensity in the NNE compared to the NT to determine if TCA fixation alters tissue-specific signal intensity as it does in microtubule proteins, and we identified that in all fixations, ECAD signal intensity was higher in the NNE than the NT. However, the difference between the two was more apparent after PFA fixation, (p ≤ 0.0001, n = 13) than in 1h or 3h TCA fixation (p ≤ 0.01, n = 13 and p ≤ 0.01, n = 12).

In contrast to the subtle changes in tissue-specific ECAD signal intensity after PFA versus TCA fixation (Fig. 5D–F, M), the NCAD signal intensity appeared to increase in the CM after TCA fixation (Fig. 5J–L). Specifically, after PFA fixation, the NCAD NT signal was significantly higher than the CM (p ≤ 0.05, n = 8), but in 1h and 3h 2% TCA fixation, the relative fluorescence intensity of NCAD increased in the CM compared to the NT, thereby reducing the difference in fluorescence intensity, after 1h (p = ns, n = 5) and 3h (p = ns, n = 5).

## 4. Discussion

Despite their widespread use, studies have shown that over 50% of antibodies fail in one or more applications (Ayoubi et al., 2023). Thus, it is vital to validate that antibodies work properly before trusting them for characterization studies or functional applications. When using a new commercial antibody, researchers will often experiment with various concentrations of the antibody but may not alter the fixation method used to process the tissue beforehand. Here, we compared the effectiveness of PFA fixation to that of TCA fixation prior to HCR and IHC in chicken embryos using multiple previously validated antibodies. We identified that the type of fixation applied affected cellular and tissue morphology, with TCA fixation resulting in larger, more circular nuclei. We also found that differences in the type and length of fixation had effects on the visualization of protein signal at the tissue-specific and sometimes subcellular level.

The morphological changes that we identified are likely due to the different mechanisms by which PFA and TCA fix tissues rather than artifacts from cryosectioning. Since all samples are fixed, imaged in wholemount, and then cryosectioned using the same methods (Fig. 1), we expect that the morphological differences are due to fixation techniques. PFA covalently cross-links molecules, stabilizing tertiary and quaternary structures of proteins and hardening the cell surface (Kim et al., 2017). We observed that in PFA-fixed chicken embryos, tissue appeared more tightly packed with denser and less circular nuclei and smaller NTs (Fig. 2). In contrast, we found that TCA fixation resulted in larger and more circular nuclei and larger NTs (Fig. 2). Rather than cross-linking proteins, TCA precipitates proteins by disrupting their encircling hydration sphere (Koontz, 2014). Unlike PFA, which maintains tertiary and quaternary structure, TCA denatures proteins to the point where their secondary and tertiary structures are lost (Koontz, 2014). The nuclei and tissue shape changes we observed may be due to this precipitation of proteins within a cell, filling up space and rounding out the nuclear and cellular membranes. However, it would be helpful to perform similar analysis using high resolution 3D imaging with light sheet fluorescence microscopy or other method in intact embryos to determine if the tissue-specific intensities change with different fixative methods.

These differences make each fixative type more ideal depending on the target epitope. Since TCA precipitates and denatures proteins, it makes hidden epitopes more accessible. In contrast, PFA is ideal for targeting structural epitopes as it maintains tertiary and quaternary structures. Here, we sought to understand how these various fixative methods affect immunohistochemical staining using antibodies for markers in multiple tissue types in chicken embryos. In contrast to PFA, which works well with short fixation times preceding antibody use, such as the 20 min used for this study, TCA results in low signal at an equivalent fixation duration (data not shown). Thus, we employed 1 h and 3 h of TCA fixation, which led to similar outcomes of cellular morphology and signal intensity when compared to each other. By using chicken embryos as our model, we were able to use commercially available antibodies we and others have previously validated (Table 2). We identified that both PFA and TCA fixation allowed us to visualize proteins in their expected locations, but we saw that some treatments altered signal intensities across tissues.

We identified a marked difference in how TCA and PFA affected the visualization of nuclear markers. Nuclear markers had weaker fluorescence signal and appeared more compartmentalized within the nucleus in TCA-fixed embryos compared to PFA-fixed embryos (Fig. 3). This result may be caused by actual subnuclear protein localization, or it may be due to the TCA precipitation of the target proteins within the nuclear compartment (Lakatos and Jobst, 1992; Rajalingam et al., 2009). In measuring fluorescence intensity of nuclear markers across nuclear membranes, we saw that for some markers (DAPI, PAX7, SOX9) there appeared to be consistent variability of the signal across the nucleus (Fig. 3W–Y) but that for others (SNAI2) there was increased variability in signal intensity across the nuclei after TCA fixation (Fig. 3Z). If this compartmentalization of the signal is biologically accurate, it is a method that could be used to visualize condensates within nuclei. Additionally, TCA fixation may allow us to compare the localization of multiple nuclear markers at once to see if their subnuclear localization differs at different phases of the cell cycle, for example. However, nuclear markers used following TCA fixation also tended to have a weaker signal compared to background, possibly due to precipitation.

PFA and TCA fixation also caused noticeable differences in the fluorescence intensity within specific tissues when used before IHC with microtubule subunits (Fig. 4). Past work showed that TUBB3 is expressed in the NT at HH8 in chicken embryos, with a stronger signal intensity at the dorsal side where the NC cells are present at HH9 (Chacon and Rogers, 2019). Here, we see similar localization in HH9 chicken embryos, but 1h TCA fixation was optimal for showcasing the increase in the NC TUBB3 signal compared to the NT signal (Fig. 4). Interestingly, TUBB2A and TUBA4A both displayed significant differences in the NNE and CM signals in the TCA fixation versus PFA fixation treatments, but in opposite directions. For TUBB2A, the NNE signal increased in relation to the CM in TCA-fixed embryos compared to PFA-fixed embryos, increasing this difference (Fig. 4T). Meanwhile, for TUBA4A, the CM signal increased in TCA-fixed embryos compared to PFA-fixed embryos, decreasing this relationship (Fig. 4U). Thus, it is critical to test multiple fixatives for markers of interest even across similar protein types, as they may enhance signal in different tissue regions. Microtubule proteins are well known for their post-translational modifications which directly affects microtubule stability (Bar et al., 2022), and it is possible that these differently modified proteins are better targeted in one fixative versus the other but this was not explicitly tested here.

We saw similar differences in cadherin protein signals using IHC between TCA and PFA fixatives. In stage HH9 chickens, equivalent to our samples, ECAD localized to the NNE, NT, migratory NC cells, and developing gut (Rogers et al., 2018). In our samples across various fixatives, we saw that the fluorescence intensity of the NNE was consistently higher than in the NT, although the NT intensity increased in TCA (Fig. 5D–F, M). Similarly, NCAD displayed the expected localization for HH9 chickens to the NT, CM, notochord, developing gut, and absence from the dorsal NT regardless of fixative type (Rogers et al., 2018). However, fluorescence intensity of NCAD in the NT was far higher than that in the CM for PFA-fixed embryos compared to TCA-fixed embryos (Fig. 5J–L, N). This result suggests that the type of fixative applied can affect the primary tissue in which a protein signal appears, and that issue may have far-reaching effects for individuals studying cell and developmental biology as those fields strongly rely on knowing spatiotemporal protein localization prior to studying protein function. Of note, although TCA fixation proved ineffective to visualize most genes, the *NCAD* gene expression was maintained strongly in the NT and weaker in the CM, which suggests that the PFA-fixed NCAD protein localization is more representative of the gene expression (compare Fig. 5G–I). However, cadherin proteins are post-translationally modified and trafficked (Abbruzzese et al., 2016; Kiener et al., 2006; West and Harris, 2016), and therefore gene expression is not always consistent with protein expression and localization. Our results have implications for characterizing new antibodies that do not have published and validated expression models.

To truly define where and when a protein is expressed and localized at subcellular- and tissue-level resolution, use of live imaging methods would be ideal. However, there are limitations to these types of analyses currently due to potential issues with protein tertiary structure changes after fused tagging with large fluorescent proteins or overexpression artifacts that can occur if proteins are introduced into an organism. Although our study provides a starting place for analyses of protein signal detection and studies of different fixation methods, we need additional technological advances like those that have been created to visualize mRNA *in vivo*. Future work may consider using methods like protein tagging paired with the electroporation of nuclear reporters or live imaging dyes to further resolve the question of protein localization in tissues and within cells.

Future studies using IHC with commercial antibodies would benefit from fixation validation in addition to traditional antibody specification validations (e.g., knockdown, overexpression, western blot) as some fixatives may improve visualization of proteins of interest. Comparing the 3h versus 1h TCA fixes to each other revealed that the 1h TCA fixation is sufficient to alter tissue morphology and to reveal additional protein signal in the tissue samples. However, fixed tissues are not living tissues and as technologies become available, it would be important to visualize these cellular events *in vivo*. It may also be beneficial to compare additional fixation techniques such as alcohol-based fixation or antigen retrieval to see if these methods replicate or improve the outcomes from PFA or TCA fixation. As displayed in this paper, the method of fixation can affect the strength of protein signals after IHC in different tissues. While PFA revealed epitopes in most tissues, TCA-mediated protein denaturation may provide access to hidden epitopes in regions of the protein of interest that are inaccessible due to PFA cross-linking (Klockenbusch and Kast, 2010; Nadeau and Carlson, 2007). Here, we only evaluated these techniques in a single organism, and we demonstrate that fixatives affect the visualization of numerous proteins in several cellular compartments. However, the type of fixative used has been found to affect cellular and tissue morphology in other systems and animals including human cell culture, goats, rats, and mice (Cox et al., 2006; Hirashima and Adachi, 2015; Paavilainen et al., 2010; Rahman et al.; Rezoana et al., 2022; Tu et al., 2011; Wang et al., 2016). The fixative type used should be optimized depending on the model system, type of protein, and expected localization. Our results demonstrate that methods can, and should, be tested for improved biological analyses and accurate demonstration of results in wholemount or in section.

## Figures and Tables

**Table 1:** Common fixative methods used for developing vertebrate embryos.

Fixative Chemical	Chicken	Zebrafish	Frogs
Aldehyde-based	(Balint and Csillag, 2007; Carro et al., 2013; Chacon and Rogers, 2019; Rogers et al., 2013a, 2013b)	(Hammond-Weinberger and ZeRuth, 2020; Macdonald, 1999; Shimomura et al., 2007)	(Acton et al., 2005; Fagotto and Brown, 2008; Kurth et al., 2010; Lee et al., 2008; Robinson and Guille, 1999; Tao et al., 2007)
Alcohol-based			(Lee et al., 2008; Ossipova et al., 2022; Ossipova and Sokol, 2021)
Acid-based		(Lasseigne et al., 2021; Macdonald, 1999; Martin et al., 2022)	
Other			Acton et al. (2005)

**Table 2:** Antibodies used in study.

Antibody target	Company and catalog #	Dilution	Isotype	Protein type/Expected localization	Immunogen
PAX7	DSHB (PAX7)	1:5–1:10	Mouse IgG1	TF/Nucleus	Recombinant protein (C-terminal region, amino acids 352–523)
SNAI2	Cell signaling, mAb9585	1:200	Rabbit IgG	TF/Nucleus	Recombinant human Slug protein
SOX9	Millipore Sigma, AB5535	1:500	Rabbit IgG	TF/Nucleus	Transcription factor SOX-9 recombinant protein epitope signature tag (PrEST)
ECAD	BD Transduction Laboratories 61081	1:500	Mouse IgG2a	Cell adhesion/Membrane	Human E-Cadherin C-terminal Recombinant Protein
NCAD	DSHB (NCAD), MNCD2	1:5	Rat IgG1	Cell adhesion/Membrane	Recombinant mouse protein domain (amino acids 308–597)
TUBA4A	Sigma, T6199 (clone DM1A)	1:250	Mouse IgG1	Cytoskeleton/Cytosol	Microtubules from chicken embryo brain
TUBB2A	Abnova, H00007280-M03 (clone 2B2)	1:250	Mouse IgG2a	Cytoskeleton/Cytosol	TUBB2A (AAH01194, 1 a.a. ~ 445 a.a) full-length recombinant protein with GST tag
TUBB3	R&D Systems, MAB1195	1:250	Mouse IgG2a	Cytoskeleton/Cytosol	Rat brain-derived microtubules

## Data Availability

Data will be made available on request.
